# Supplementary material for: Implementation and prospective real-time evaluation of a generalized system for in-clinic deployment and validation of machine learning models in radiology
Source: PLOS Digit Health. 2023 Aug 21;2(8):e0000227. doi: 10.1371/journal.pdig.0000227 (PMC10441783; doi:10.1371/journal.pdig.0000227)
Supplement: Attachment — Submitted filename: Response to Reviewers.docx [file pdig.0000227.s001.docx]

PLOS DH Review and Editorial Team,

Thank you for taking the time to consider and review our paper, "Implementation and prospective real-time evaluation of a generalized system for in-clinic deployment and validation of machine learning models in radiology." We appreciate the reviewers’ comments, and have revised our manuscript to address the points that were raised (please see below). We look forward to hearing back from the PLOS DH team.

Thanks,

James Hawkins

James Hawkins

Lead ML Operations Engineer

Intelligent Imaging Computational Core, UCSF Dept. of Radiology and Biomedical Imaging

e: [james.hawkins@ucsf.edu](mailto:james.hawkins@ucsf.edu)

## Reviewer #1

My concern is that with open source software the issues become apparent only with the passing of time. The risks of software becoming outdated or “orphaned” software, untracked dependencies etc. may pose a risk down the line. Often it can take up to a year for vulnerabilities to show up in open source based software.

Is there a DevSecOp built into the process? and does the cost -benefit analysis account for the time/expertise to keep the pipeline secure/stable. I would urge the authors to write a short status report in 18 months timeframe to report on vulnerabilities(if any).

The section “Building an open-source based inference system” has been added to the Discussion. It is a substantial addition, but both reviewers asked for clarification on the decision to build on open-source as opposed to purchasing a commercial solution. The subsection “Software vulnerabilities and system security” covers the topic of security and how we utilize UCSF’s enterprise vulnerability assessment platform. We also mention at the end of the “Pipeline development and deployment” subsection of “Materials and methods” that our system underwent the UCSF IT Security Risk Assessment process, which reviews all IT projects for data and security risks.

Materials and methods -This section is clear and well explained. My only suggestion for this section is to release some in-house development code via Github to best estimate the efforts for a software team that is hoping to learn from the process and estimate the learning time.

NVIDIA provides an “Operator Development Kit,” which covers building Clara Deploy operators from scratch. This is now covered in “Materials and methods,” line 224. The link to documentation and downloadable source code is in reference 35.

Discussion section - Raises some valid points especially about open source software. Once again , it would be helpful to review the system reliability a year away from deployment.

A paragraph has been added to the end of the “Results” section, covering system performance and reliability. Beginning at the time of initial provisioning, we cover the total exams processed and the current daily/weekly workload. The system has been operational for 2 years without negatively impacting clinical systems and without the stability (or lack-there-of) in any open-source dependencies causing reliability issues.

## Reviewer #2

The main point I would like to see better clarified is the choice landscape of cloud vs on-prem: costs, IRB constraints, time requirements. At each stage and for each element of the system, please state whether it is in-cloud or on-prem, whether this by choice or effectively required, and the pros and cons. We have found this to be a major issue to consider. If you can, please address the question of the varying levels of possible control of cloud resources, and how these can meet IRB requirements.

The system described is entirely on-premises, and this has been more clearly emphasized throughout the manuscript (lines 88, Fig 1 caption line 97, line 138, line 166). The subsection “Deploying on-premises” in the Discussion’s new “Building an open-source based inference system” section covers in more detail the rationale for building on-premises and also why on-prem is a good choice for projects matching our requirements. We mention that all systems processing sensitive data need to undergo institutional security review, whether internally hosted or in the cloud, and that HIPAA Business Associate Agreements can be established with cloud providers to allow the transfer of sensitive data.

56: typo with period and citation (also in other spots). Also, the citations seem to lack a preceding space.

Citation formatting across the document was fixed with preceding spaces and punctuation following the enclosing brackets (referencing the sample manuscript from the submission guidelines).

62: stray "and" before "effectiveness"

The sentence has been reworded for clarity (line 63).

86: What is Nvidia Clara Deploy - brief description please. In general, brief orienting descriptions inline help the reader immensely.

Thank you for the suggestion. The following explanation was added (see “Data Flow,” line 88): “NVIDIA Clara Deploy [14], a software platform for deploying ML pipelines.”

86: What does / is XNAT? as above.

The following explanation of XNAT was added (see “Data Flow,” line 89): “Results are exported to a dedicated instance of the XNAT web application, running on the same host. XNAT stores and displays inference results separate from the clinical record, which Clinicians can still access from a PACS workstation in the reading room or other UCSF computers via a browser.”

100: What does OHIF mean?

OHIF is the name of the organization that created the image viewer used in XNAT. This caption has been updated to use the wording “XNAT’s image viewer”, and OHIF specifically is mentioned as a medical image viewer, with citation, in line 173.

Table 1: Combining cols 1 and 2 would simplify the table structure

Column 1 indicates the validation use case, whereas column 2 presents an example of a DICOM routing configuration parameter used for that use case.

133: Regularize spelling of Nvidia across paper

We have confirmed all text references are spelled consistently as “NVIDIA”, apart from “nvidia” appearing in URLs.

133: Please give brief description of Triton. In particular, is it run in the cloud? This is an example of the main point described above.

The following description of Triton has been added in-line (see “AI Inference Framework,” line 158): “NVIDIA’s Triton Inference Server [22], an AI inference application optimized for GPU performance and bundled with Clara Deploy. The Triton container is managed by Clara Deploy, and is used by pipelines to perform inference, loading models in and out of GPU memory when needed.”

144: what is REST?

The following text description has been added about REST, with more information in the existing reference (see “Delivery of Results,” line 172): “REST Application Programming Interface (API) [23], which allows pipelines to store and modify data via standard web protocols.”

Somewhere in this section: Please discuss where data is stored. Especially, are there separate channels for identified and de-identified patient data? How does data storage interact with IRB requirements, and with upload/storage costs?

Along with the on-premises discussion in the section “Deploying on-premises,” the following text has been added/modified to highlight where that the data is stored in XNAT on the inference server:

- (“Abstract,” line 27): “with results and clinician feedback stored in a customized XNAT instance, separate from the clinical record but linked from within PACS.”,
- (“Data Flow,” line 89): “Results are exported to a dedicated instance of the XNAT web application, running on the same host. XNAT stores and displays inference results separate from the clinical record, which Clinicians can still access from a PACS workstation in the reading room or other UCSF computers via a browser”,
- (“Delivery of Results,” line 181): “The XNAT application and its Postgres [25] database are each run inside Docker containers deployed on the inference server,”

Table 3, and line 187: Clara Deploy DICOM segmentation writer: Does this require collaboration with Nvidia? If so, was this by choice, and are there other options?

The authors clarified that modifications were possible because the Clara platform is open source (see “NVIDIA Clara Deploy operators,” lines 222 and 226): “These modifications are possible because the Clara operator source code is open and accessible, removing the need to rely on NVIDIA engineers to implement feature requests.”

183: Python (capitalized)

The authors verified that all instances of Python are capitalized.

219-220: is clinician feedback text only, or is for example segmentation correction on images possible? please discuss the customizability of this piece.

Please see the description of forms and image editing capability (“Management of Results,” line 244): “Each validation study has a custom feedback capture form integrated with the results, with freeform text inputs, dropdowns, and other standard HTML inputs. Clinicians can make non-destructive edits to ML segmentations, with edited segmentations saved back into XNAT and linked to the source exam.”

243 ff: Is on-site storage effectively required (for the reasons stated later)? If yes, please state this explicitly.

The system is on-premises (see previous comments), and text was added to state the requirement for this project (“Deploying on-premises,” line 433): “For this project, leveraging cloud-based infrastructure was not a viable option…”

298: "in separate papers" is clearer (ie not separate subsections)

The text was changed to read (“Results,” line 345): “in separate papers.”

316: what/who is the subject of the verb "supported"

The text was modified to read (“Results,” line 364): “Results and findings from this validation study were reviewed by ci^2^’s Clinical Deployment committee (see Discussion: Governance and Validation Criteria), which supported adoption of the pipeline for routine clinical use at UCSF.”

367: normalize spellings of ci^2 (at least 3 versions in paper)

The authors verified that all instances are spelled constituently as “ci^2^”.

382 ff: what are the general constraints on ability to import ML frameworks, eg Python? PyTorch? Matlab? Matlab bundled as an executable only? How much effort is required, eg is it just a case of configuring a docker, or is it more work. This is an important question for ML readers

A statement was added defining requirements for incorporation of models from other frameworks (see “Deployment Framework Flexibility,” line 506): “Clara Deploy supports running inference with models from external frameworks, provided inference can be run within a Python executable.”

402 - 403: I believe strong and opposite arguments can be made (eg by our IT department) for choosing software with enterprise support - can you please discuss the pros and cons of open-source more deeply?

The rationale for choosing to build upon open-source software over commercial options is discussed in the added section “Building an open-source based inference system,” paragraph 1.

411 -452: Awesome content. Perhaps it be placed earlier in the paper, eg introduction/background. These issues are fundamental and presumably drove many of your architecture choices. In any case, readers would benefit by having these issues well in mind as they read about your proposed solution.

The fact that results are stored separate from the clinical record, but accessible from within clinical resources like the PACS, has been emphasized in:

- (“Abstract,” line 27): “with results and clinician feedback stored in a customized XNAT instance, separate from the clinical record but linked from within PACS.”,
- (“Data flow,” line 90): " XNAT stores and displays inference results separate from the clinical record, which clinicians can still access from a PACS workstation in the reading room or other UCSF computers via a browser."
- (“Delivery of results,” line 178): “Using XNAT as a data store is a key component of this system, as validation study results are separate from the clinical record but can still be integrated within a clinician’s workflow.”

464: "will ultimately support any...": this is a bold claim. Is it accurate as stated?

Agreed. The sentence was modified to read (“Conclusion,” line 586): “The server and network architecture put in place is positioned to adapt to support new standards-compliant ML model deployment frameworks used down the road.”

476 - 477: See note to 402-403

As above, see section “Building an open-source based inference system.”

Multiple figures: in my printout they are landscape, requiring turning the paper 90 degrees. How will they be presented in the final version, and will they be legible if shrunken? Perhaps they could be better restructured to be vertical, to better use a page and allow larger fonts.

The data flows and orientations of the diagrams suit a left-to-right horizontal presentation. Higher-resolution versions of all figures have been generated to increase legibility. Additionally, Figure 2 has been split into Figures 2, 3, and 4 to allow for larger individual images. These figures take advantage of PLOS DH being a digital-first publication, where embedded images can be clicked-on and zoomed in-browser. The images have all been passed through PLOS’ PACE tool, which does adjust the size and DPI from the original inputs. Captions have also been updated for more clarity on figure contents.

Figure 1: (a) describe Hopper (b) are inference results stored in the inference server, or is this just an interface area? See comments about storage, privacy, etc above. Are they stored as dicoms, or different file types? Are they lightweight (low space demand)?

The caption for Figure 1 has been updated to read: “**Fig 1: High-level system architecture and data-flow diagram of on-premises clinically integrated inference validation service.** From top left, clockwise: 1) Clinical imaging modalities and resources networked via DICOM communication protocols. 2) AI inference server running Clara Deploy and XNAT, connected to imaging resources via DICOM. 3) A “hopper” server that gates access to clinical infrastructure, used by system admins and engineers to connect to the inference server from within the radiology network via ssh. 4) Clinicians access the results stored in XNAT from the reading room and other computers within UCSF.” Comments on storage and privacy are addressed above.

Figure 4: bad quality/unreadable text - is a higher resolution available?

The descriptive text in Figure 6 (originally Figure 4) has been enlarged from font size 18 to 24, and the figure re-exported at a higher resolution.

Figure 5: Ditto - is a higher resolution version available?

The screenshot was re-taken and exported at a higher resolution (now Figure 7).
